# Accountability, autonomy, equity, and trust in selected healthcare systems: a comparative analysis

**DOI:** 10.1186/s13584-025-00736-x

**Published:** 2025-12-17

**Authors:** Tal Sigawi, Doron Yosef, Orna Tal

**Affiliations:** 1 Bar Ilan University, Ramat Gan, Israel; 2 Faculty of Medicine, Department of Medicine, The Hebrew University, Hadassah Medical Center, Jerusalem, Israel; 3 Leumit Health Services, Jerusalem, Israel; 4 Shamir Medical Center, Asaf Harofeh, Beer Yaakov, Israel; 5 The Israeli Center for Emerging Technologies (ICET), Zrifin, Israel

**Keywords:** Accountability, Autonomy, Equity, Trust, Satisfaction, Health policy, Health system comparison

## Abstract

**Aim:**

Healthcare systems struggle to balance priorities within complex settings. This analysis aims to identify cross-national insights and trade-offs in healthcare policy by examining how five high-income countries navigate accountability, autonomy, equity, trust, satisfaction, and system effectiveness – competing parameters that shape system performance and public experience.

**Methods:**

A structured comparative analysis was conducted across five national healthcare systems: Israel, the Netherlands, Germany, the United Kingdom (UK), and the United States (US). Publicly available literature, including peer-reviewed studies, policy reports, and population surveys, was synthesized using a narrative approach due to variation in data definitions and context.

**Results:**

The Netherlands and Germany demonstrate relatively balanced performance across all parameters, supported by regulated insurance models. Despite universal coverage, the UK system faces sustained access failures and eroding public satisfaction. The US underperforms in equity and satisfaction but demonstrates strengths in specific clinical domains. Israel combines strong statutory coverage with growing reliance on supplementary and private insurance, raising concerns about long-term equity and regulatory coherence.

**Conclusions:**

Each system reflects different strengths and trade-offs across the examined dimensions. The findings highlight structural tensions between autonomy and accountability, equity and access, decentralization and fragmentation, and public versus private provision that shape overall system performance. These insights are relevant for health systems seeking to enhance care delivery in an effective and patient-satisfying manner, and they support cross-national dialogue on designing resilient and equitable health systems.

## Introduction

Healthcare systems worldwide vary widely in their structure and performance. Healthcare leaders across systems struggle to address the interplay between system-level *accountability* and patient *autonomy* – two core principles central to delivering effective and equitable care. These foundational concepts are intertwined with other key indicators of performance, including *equity*, *trust*, *satisfaction*, and overall *system effectiveness*. Together, these six parameters offer a structured lens for cross-country comparison.

This comparative analysis focuses on five national healthcare systems: Israel, the Netherlands, Germany, the United Kingdom (UK), and the United States (US), representing a range of governance models and institutional arrangements. These systems offer diverse strategies for managing regulation, funding, and patient involvement, and provide relevant insights for global policy learning. By considering both system-level design and patient-centered experiences, this framework evaluates how each system addresses shared challenges.

While performance across parameters is influenced by broader cultural, political, and historical contexts, this analysis focuses primarily on measurable system characteristics. It assesses how accountability, autonomy, equity, trust, satisfaction, and system effectiveness manifest across the five selected healthcare systems, aiming to generate comparative insights that may inform future health policy and promote cross-national discussion.

## Methods

### General approach and data collection

A structured comparative analysis was conducted based on publicly available literature, including OECD reports, peer-reviewed publications, national research institute outputs, and government documents. Data were compiled for five countries: Israel, the Netherlands, Germany, the UK, and the US. The analysis was systematically evaluated across the six parameters (accountability, autonomy, equity, trust, satisfaction, and system effectiveness) to ensure consistency and comparability, encompassing institutional design, performance, and patient experience. Where possible, standardized indicators were used for comparison. However, due to differences in definitions and measurement standards across countries, most parameters required contextual interpretation based on narrative synthesis.

### Parameter definitions

While parameter definitions may vary across healthcare systems, the following operational definitions were used in this analysis to standardize comparisons:

#### Accountability

The responsibility of governments and insurers to ensure high-quality, accessible care and to be subject to oversight or corrective actions when standards are not met [1, 2]. From the perspective of the regulators or healthcare leadership, this includes clinical performance, regulatory oversight, transparency, and responsible resource allocation, all understood within a broader framework of social responsibility [3]. 

#### Autonomy

The capability of a patient to make voluntary, informed decisions regarding healthcare, particularly in choosing a physician or care provider [4]. This focus on provider choice reflects a core value that shapes the therapeutic relationship, trust in care, and perceived quality [5–7]. In some health systems, patients may also choose their insurer or the coverage plan, choices that are often shaped by financial, administrative, or regulatory constraints, such as affordability, risk coverage, or benefit restrictions [8]. 

#### Equity

The process of eliminating systematic, avoidable, and unjust differences in access to healthcare services across population groups, regardless of geographic location, socioeconomic status, or cultural background [9–11]. This includes both intra-system differences (e.g., geographical or socioeconomic disparities within public systems) and inter-system disparities between public and private coverage, which may drive differential access and outcomes.

**Trust** 

Public confidence in the sustainability, reliability, and fairness of the healthcare system, encompassing expectations that the system will consistently deliver competent, ethical, and equitable care over time [12–14]. Trust operates at multiple levels: interpersonal (e.g., physician–patient), institutional (e.g., provider networks), and systemic (e.g., government stewardship) [13, 15]. Trust is both an outcome of system performance and a determinant of health-seeking behavior, compliance, and engagement [13].

**Satisfaction**

The degree to which individual patients or the public report that their experiences with the health system meet their expectations, particularly regarding affordability, accessibility, communication, and service quality [16–18]. Satisfaction reflects perceived system performance, which is shaped by direct experiences (e.g., recent care episodes), indirect experiences (e.g., friends and family), and broader societal context (e.g., media, policy debates, cultural norms) [18–20]. 

#### System effectiveness

The ability of a healthcare system to achieve desired clinical outcomes, such as reduced avoidable mortality, lower rates of preventable hospitalizations, and increased life expectancy, relative to resource investment. While this outcome-based definition does not capture all components of effectiveness, it focuses on the efficiency with which health systems translate financial and structural inputs into measurable improvements in population health status. This definition is intentionally limited to outcome-based measures, not encompassing all components of effectiveness, such as structural quality (e.g., infrastructure, workforce) or process quality (e.g., adherence to clinical guidelines) [21–24]. Related domains, including access, trust, and patient satisfaction, are addressed separately within this framework, as they represent distinct but complementary aspects of system performance.

## Results

### A comparison of selected healthcare systems

The five healthcare systems examined in this review represent a wide spectrum of institutional models and policy strategies. The following sections explore how these health systems organize the provision of care, focusing on the roles of accountability and autonomy and their relationship to the observed patterns of equity, trust, satisfaction, and system effectiveness.

1. **Israel**

#### Overview

The Israeli healthcare system is predominantly public, providing universal coverage guaranteed through the National Health Insurance Law, funded by government allocations and progressive health taxes [25]. Four not-for-profit health maintenance organizations (HMOs) deliver care under national regulation [25]. Nevertheless, private spending is substantial, as 32.1% of total healthcare expenditures are out-of-pocket, among the highest in the OECD, largely due to widespread supplementary and commercial insurance plans [26, 27]. This dual structure, combining strong public coverage with a significant private component, shapes both access and system performance.

#### Accountability

The government plays a central role in ensuring access to healthcare services, primarily through the regulation and funding of the HMOs that provide mandatory universal coverage [25]. Public oversight mechanisms, such as periodic reforms via the Economic Arrangements Law and monitoring by the Ministry of Health, emphasize transparency, resource stewardship, and institutional responsiveness [28–31]. 

#### Autonomy

Autonomy is primarily reflected in the ability to choose both the insurer and the physician, the latter mostly through private mechanisms. In recent years, autonomy has expanded through reforms that broaden provider choice within the public system. The Economic Arrangements Law enhanced patient flexibility in selecting providers, while the 2024 Health Information Mobility Law improved care coordination and supported more informed decision-making [28–31]. 

#### Equity

Despite universal coverage, disparities persist across socioeconomic and geographic lines. Peripheral regions face workforce shortages, and lower-income households are more likely to experience barriers due to high out-of-pocket costs [26]. Although over 80% of the population holds supplementary insurance to address concerns about access and timeliness, this reliance risks deepening inequality for those unable to afford it [26]. While the core benefits package is uniformly available within the National Health Insurance Law framework, stratification emerges in access to non-urgent services, specialist consultations, and private care [25]. Equity in Israel is thus challenged by both structural limitations within the public system and the growing influence of private financing, even as the system remains grounded in a universal model.

#### Trust

Public trust reflects both institutional resilience and specific areas of concern. In a 2022 national survey, 62% of respondents expressed trust in the system’s ability to deliver appropriate care, but only 42% felt confident that treatment costs would be covered [27, 32]. Nonetheless, trust is not uniform across all system levels. Low HMO switching rates (2–3% annually) and long-term membership patterns suggest strong trust in individual insurers [30, 33]. This layered dynamic indicates that while individuals maintain confidence in their direct relationships with providers and HMOs, broader policy and financing structures remain sources of skepticism. Recent reforms aimed at promoting transparency and reducing redundancy in supplementary insurance may influence system-level trust over time [34]. 

**Satisfaction**

Public satisfaction has grown in recent years. A 2022 Brookdale survey reported that 68% of respondents were satisfied with the healthcare system overall, up from 58% in 2018 [32]. Satisfaction with specific HMOs was even higher, reaching 88%, highlighting positive day-to-day experiences. Still, nearly 35% of respondents reported forgoing care due to long waiting times, suggesting that dissatisfaction is driven more by system responsiveness than by service quality [26, 32]. These findings point to a stratified perception of satisfaction across the system hierarchy, as people value the care they receive but remain frustrated by broader structural barriers.

#### System effectiveness

Health indicators place Israel among the top healthcare systems globally, demonstrating remarkable performance despite a limited budget. Israel reports the highest average life expectancy at birth (82.6 years) and the lowest infant mortality rate (2.8 per 1,000 live births) among the countries analyzed​ [26]. In 2022, Israel’s healthcare expenditure was 7.4% of GDP, below the OECD average of 9.2% [27]. However, system strain is evident in long waiting times, geographic service disparities, and increased reliance on private care, which may undermine sustainability [26, 32]. Maintaining system effectiveness will require ongoing investment in infrastructure, workforce, and equitable access.

#### Summary

Israel’s healthcare system exemplifies a balance between strong public foundations and increasing private influence. It achieves strong health outcomes on limited resources, but ongoing disparities in access and growing reliance on private financing underscore the system’s vulnerability and the need for continued adaptation.

2. **The Netherlands**

#### Overview

The Dutch healthcare system ensures universal access through a statutory insurance model that combines robust national oversight with regulated market competition [35, 36]. All residents are required to purchase insurance under the Health Insurance Act for routine medical services and the Long-Term Care Act for chronic care and nursing support [37]. While access and financing are regulated at the national level, service delivery is handled by a wide network of private providers. Most general practitioners (GPs), hospital-based physician groups, and health insurers operate as private for-profit entities, though they are subject to strict regulation [35]. 

#### Accountability

Accountability in the Dutch system is anchored in centralized regulation over a competitive market, where private actors operate under strict national oversight [37, 38]. The government mandates a uniform benefits package and requires all insurers to accept applicants regardless of health status [37]. Risk equalization redistributes funds based on enrollee characteristics, limiting incentives for risk selection and promoting fairness. National framework agreements between government, insurers, and providers set targets for cost control, service quality, and innovation. Supervision is enforced by the Dutch Health Care Authority and the National Health Care Institute, which monitor spending, quality, and adherence to national standards [36]. This structure presents the central role of the private sector in service delivery, managed through a strong public governance framework.

#### Autonomy

Residents are free to switch insurers annually, and they retain the right to choose providers [35, 38]. However, selective contracting, where insurers limit networks to certain providers, can restrict meaningful choice, especially for hospital or specialist services [36]. Thus, while the formal mechanism supports patient choice, actual experience is shaped by availability and insurer-defined networks.

#### Equity

Equity is supported by mandatory coverage, income-adjusted premiums, and low rates of unmet health need, reported at 0.2% [27, 38]. Public funding accounts for approximately 85% of health expenditure, and out-of-pocket costs are moderate (9.3% of total healthcare spending). However, a mandatory annual deductible may burden low-income individuals. Additionally, workforce shortages in primary care and selective contracting disproportionately affect rural populations, although geographic inequalities in health resources remain relatively minor [27, 35, 37, 38]. Hence, while the system performs well on equity overall, structural and regional disparities may influence access.

#### Trust

Trust in the healthcare system is reinforced by strong regulation and mandatory coverage. Transparent pricing and comparative provider data enhance institutional trust. High trust is reflected in broad public satisfaction, minimal reported barriers to access, and relatively low annual insurer switching rates of 6–7% [35]. 

**Satisfaction** Public satisfaction is consistently high. In the 2023 OECD report, 83% of respondents expressed satisfaction with the availability of quality healthcare [27]. However, this satisfaction may be moderated by GP shortages and selective contracting practices that can limit patient choice [35]. 

#### System effectiveness

The Dutch healthcare system delivers strong outcomes alongside high investment. Average life expectancy exceeds the OECD average (81.4 vs. 80.3 years), and infant mortality rates remain low (3.3 deaths per 1,000 live births). In 2022, health expenditure reached 10.2% of GDP, above the OECD average, primarily reflecting the cost of comprehensive services and extensive long-term care coverage [35, 38]. 

The 2022–2023 Integrated Care Agreement reform, signed by healthcare sector stakeholders, aims to maintain high-quality, accessible services [35, 36]. It shifts the focus from illness to health, emphasizes preventive medicine, supports vulnerable individuals, improves workforce distribution, and reduces administrative burdens, aiming at cost containment and improved efficiency.

#### Summary

The Netherlands exemplifies a regulated insurance-based model that balances market mechanisms with central supervision to achieve high health system performance [35, 36]. While equity and satisfaction remain strong, emerging challenges related to workforce shortages will require targeted policy responses to preserve trust and effectiveness.

3. **Germany**

#### Overview

In Germany, a large statutory health insurance (SHI) sector covering approximately 89% of the population coexists with a private insurance option that covers around 11%, primarily higher-income or self-employed individuals [39, 40]. The SHI system is publicly funded through income-based taxes, which are pooled and redistributed among 96 sickness funds via a central health fund based on risk adjustment [39, 40]. 

#### Accountability

Accountability is shared across federal, state, municipal, and non-governmental actors. The Federal Ministry of Health defines the national legal framework and policy, while the Länder (states) plan hospital capital investments, including infrastructure and equipment [40, 41]. However, many publicly owned hospitals are municipally owned and managed, with local governments overseeing day-to-day clinical operations. This dual sub-national governance model (state-level capital investment and municipal operational control) reflects a deliberate form of decentralized responsibility, shaped by Germany’s federal structure, regional needs, and population size. Sickness funds and private insurers operate under national regulations to contract with providers and manage financing [39]. Although accountability is dispersed across multiple actors, it remains functionally aligned through national health legislation, quality monitoring, and system-wide standards.

#### Autonomy

Germany offers high patient autonomy, with individuals able to access most specialists directly without formal gatekeeping [42]. Both statutory and private insurance holders enjoy broad provider choice. However, autonomy is experienced differently across insurance types, as privately insured patients often benefit from faster access and fewer administrative barriers [39, 40]. While the system values patient choice, it is associated with increased specialist use and rising costs, prompting policy debates about balancing autonomy and efficiency [43]. 

#### Equity

Germany’s healthcare system performs well on many equity indicators, particularly within the SHI, ensuring universal coverage, income-based contributions, and broad service access. According to OECD data, only 0.3% of low-income individuals report unmet medical needs [39]. Cost-sharing mechanisms are used, with modest out-of-pocket payments covering 11% of healthcare expenditures, primarily for long-term care, pharmaceuticals, and outpatient services [40]. However, equity concerns persist due to differences between the statutory and private sectors [39, 40, 44]. While a majority of the population believes that all insured persons have the same access to medically necessary care, only a minority agrees that statutory and private insurance patients receive the same quality of care [44]. These disparities have raised concerns about a two-tier system, where access and quality vary by income or employment status. Interestingly, in 2005, Germany introduced a long-term care insurance supplemental charge for childless adults over the age of 23, based on the assumption that they may place a greater burden on the system. This policy illustrates how financing structures can incorporate demographic incentives to promote equity [45]. 

#### Trust

Most patients express strong confidence in their physicians and providers [46]. However, Trust in the healthcare system overall is more ambivalent, with perceptions differing by insurance type [47]. 

**Satisfaction** In a 2023 OECD survey, 85% of respondents were satisfied with healthcare availability [27]. Satisfaction tends to be higher among privately insured patients, who face fewer delays and restrictions [47]. 

#### System effectiveness

Life expectancy (80.4 years) is close to the OECD averages, and infant mortality rates are lower, at 3.0 deaths per 1,000 live births [37]. In 2022, health expenditure reached 12.7% of GDP, the highest in Europe [37]. Effectiveness is constrained by systemic inefficiencies, particularly overuse of inpatient care, poor coordination across sectors, and regional variation [39, 40]. Recent reforms targeting hospital financing and staffing aim to address these issues [39]. 

#### Summary

Germany’s healthcare system offers robust access, patient autonomy, and consistent performance on key health indicators, operating under a decentralized but functionally coordinated structure. However, its structural complexity, multiple actors, and disparities between insurance plans challenge equity and efficiency. Addressing inefficiencies, while preserving the strengths of regional accountability, will be key to sustaining performance and public confidence.

4. **The United States**

#### Overview

The US healthcare system is institutionally complex, comprising public programs, private insurers, nonprofit institutions, and for-profit providers [48]. While private insurance covers the majority of the population, federal programs, such as Medicare, Medicaid, and the Veterans Health Administration (VA), serve vulnerable groups, including older adults, low-income individuals, and veterans [48, 49]. According to the US Coverage Report, 92% of the population had health insurance in 2023–65.4% with private insurance and 36.3% with public plans [49]. However, coverage quality, provider access, and cost-sharing obligations vary widely by insurance type, income, and geography.

This structural complexity reflects the country’s constitutional framework and historical evolution. Decentralized authority spans federal, state, and municipal actors, alongside a wide array of nonprofit and for-profit institutions. Religious and cultural factors also shaped institutional development, as reflected in the establishment of Catholic and Jewish hospitals in the 19th and 20th centuries [50, 51]. Later, the expansion of for-profit hospital chains in high-growth regions reflected capital availability and regional demand [52]. These dynamics illustrate that decentralization in the US is an adaptive response to a large, heterogeneous society.

#### Accountability

Accountability mechanisms differ substantially between public and private sectors. Programs like Medicare, Medicaid, and the VA operate under structured federal or state supervision, with defined benefits, quality reporting, and regulatory controls. In contrast, private insurers, covering most working-age adults, display considerable variation in benefit design, provider networks, and administrative practices [48, 49]. Medicare enrollment has expanded with population aging, currently covering 99.1% of seniors, while uninsured rates among younger adults remain high [49, 53]. 

Further complexity arises from the heterogeneity of healthcare providers, limiting national coordination and contributing to variable performance across regions and populations [48]. Without national standards for access and interoperability, structural diversity may evolve into fragmentation, undermining consistency and equity. National initiatives such as the Affordable Care Act (ACA) have promoted transparency and performance-based incentives, but the absence of unified oversight limits system-wide accountability [54]. 

#### Autonomy

The US system emphasizes formal autonomy, offering individuals a broad choice of insurers and providers. However, autonomy is often constrained by cost and plan design. High deductibles and significant out-of-pocket expenses limit meaningful access, especially for lower-income populations [55]. Public insurance programs may impose further restrictions through managed care contracts and regional provider shortages [48, 49]. While consumer choice is a defining feature of the system, financial and structural barriers frequently diminish the scope of actual decision-making.

#### Equity

Despite publicly funded programs for vulnerable groups, the US exhibits significant inequities in access and outcomes. Insurance is closely tied to employment, and uninsured rates remain higher among racial minorities and low-income groups, with 21.2% of adults in the lowest income quintile lacking insurance compared to 3.8% in the highest [48, 49]. A growing body of research highlights how systemic racism, cultural bias, and chronic exposure to discrimination adversely affect mental and physical health outcomes among marginalized populations [9, 56, 57]. These disparities reflect not only differential access to care, but also broader social determinants, such as housing, education, environmental exposures, and stress, accumulating across the life course. Despite increasing coverage, high out-of-pocket spending, accounting for 11.1% of total health expenditures in 2022, poses a financial burden and restricts access for many [48]. Public programs like Medicaid and the Children’s Health Insurance Program (CHIP) aim to reduce these gaps, but eligibility and provider access vary by state [58]. Furthermore, many lost eligibility for Medicaid in 2023 due to post-COVID policy rollbacks [48, 53]. While public hospitals, nonprofit institutions, and legal mandates for emergency care provide some safety-net access, these do not fully compensate for the systemic disparities embedded in financing, service availability, and insurance design of US healthcare.

#### Trust

Trust in the US healthcare system is uneven and fragile. Patients often express strong confidence in individual providers, especially personal physicians. However, systematic trust is weakened by concerns about affordability and dissatisfaction with federal supervision [59]. 

**Satisfaction**

Public satisfaction with the US healthcare system has declined. A 2024 Gallup poll reported that only 44% of Americans rated healthcare quality as “excellent or good”, the lowest level recorded since 2001 [60]. Dissatisfaction is especially related to unaffordability, long waiting times, and insurance complexity [60]. High costs and administrative burden continue to shape negative public sentiment.

#### System effectiveness

With total health expenditure reaching 16.6% of GDP in 2023, the US invests more in healthcare than any other country. Yet, population-level outcomes lag behind peers [27, 48]. Life expectancy remains below the OECD average at 76.4 years, while infant mortality is higher, at 5.4 deaths per 1,000 live births [27]. These gaps reflect not only health system unavailability but also broader social factors, such as sedentary lifestyle, gun violence, and income inequality, that extend beyond the reach of medical care [61, 62]. 

Nonetheless, the US excels in specific domains, leading in biomedical innovation, advanced technology adoption, and high-end specialized care. Major academic centers and private systems deliver high-quality services, particularly for complex or rare conditions. System effectiveness, thus, reflects a tension between world-leading clinical capabilities and structural barriers that limit equitable access and care continuity [63]. 

#### Summary

The US healthcare system combines clinical innovation and excellence in select domains with persistent inequity, cost inefficiency, and gaps in outcomes. While it offers formal autonomy and technological leadership, these strengths coexist with variable access and inconsistent population health performance. The system’s shortcomings do not stem from decentralization or private-sector involvement per se, but more significantly from the lack of unified oversight, national standards, and coordinated policies. Addressing these challenges will require reforms that not only expand coverage and control costs, but also align the system’s diverse components around shared national health goals.

5. **The United Kingdom**

#### Overview

Healthcare in the UK is delivered primarily through the National Health Service (NHS), a publicly funded and centrally managed system that provides universal coverage and healthcare services free at the point of use [64]. However, private healthcare spending has been increasing, particularly for elective procedures, due to long waiting times in the public sector, with around 10% of the UK population currently holding private insurance [64]. 

#### Accountability

The NHS and the Department of Health and Social Care feature centralized accountability by setting budgets, defining service priorities, and monitoring performance [64]. Formal accountability mechanisms include public reporting, national performance targets, and independent supervision by the Care Quality Commission (CQC) [64]. This centralization supports national standards and strategic coordination, as well as political responsibility that may hinder effective delivery of care. The COVID-19 pandemic exemplified this dynamic, with national coordination enabling rapid vaccine deployment, while shortcomings in testing, preparedness, and workforce resilience were linked to centralized governmental decision-making [65, 66]. 

#### Autonomy

Most individuals are assigned to GPs based on geographic catchment, and specialist access generally requires GP referral [64]. This gatekeeping model promotes continuity and efficient use of resources but restricts direct access to specialists. Provider choice is also shaped by NHS commissioning and regional capacity. While patients may express preferences, their autonomy is constrained by locality and availability [64]. Greater flexibility exists for those who use private insurance or pay out of pocket, though this applies to a small portion of the population [64]. Overall, the NHS prioritizes equity and system-wide coordination over expansive patient choice.

#### Equity

The NHS was founded on principles of equity and universal access. All legal residents are entitled to free care, regardless of income or employment status [64]. While the NHS guarantees free care in principle, accessibility is increasingly limited by system strain and resource shortages. Growing delays in access to primary and emergency care have significantly affected service delivery [67]. Wait times for GP appointments are rising, emergency departments are routinely overcrowded, and ambulance services face severe pressure, with life-threatening response times frequently exceeding national targets [67]. Over recent decades, NHS-funded admissions have more than doubled, yet capacity has not kept pace. As a result, patients able to afford private services often receive faster care, with waiting times for private providers significantly shorter than those within the NHS, indicating socioeconomic disparities [68, 69]. Additionally, the UK faces criticism for limited access to novel therapies, as cost-effectiveness assessments by the National Institute for Health and Care Excellence (NICE) may delay or restrict even life-saving treatments [67]. These trends highlight that formal equity in coverage does not ensure fair access to timely and effective care, and may compromise patients’ outcomes.

#### Trust

Trust in the NHS has traditionally stemmed from its universality and symbolic role in British national identity. However, confidence has declined in the face of persistent access problems, staffing shortages, and visible service failures [64, 70]. Recent surveys indicate that although 72% of the population continues to value the NHS as a core part of British society, around 26% question its viability in its current form, citing resource constraints and growing demand [70]. These shifts reflect rising public concern over the system’s ability to deliver on its founding promises.

**Satisfaction**

Public satisfaction with the NHS has declined sharply in recent years [27]. In the 2023 British Social Attitudes survey, satisfaction dropped below 30%, the lowest recorded in over four decades [71]. Key concerns included long waiting times, difficulty accessing GPs, and workforce shortages [64]. While many still support the NHS’s founding principles, this symbolic alignment increasingly coexists with frustration over deteriorating service quality and unmet needs. The widening gap between institutional ideals and patient experience underscores growing public disillusionment with system performance.

#### System effectiveness

Life expectancy stands at 80.4 years and infant mortality at 4.0 deaths per 1,000 live births, both comparable to OECD averages [27]. In 2022, healthcare spending reached 11.3% of GDP, while out-of-pocket spending remained among the lowest in the OECD [64]. However, recent increases in expenditure have primarily targeted pandemic recovery and backlog reduction, rather than systemic capacity expansion. As a result, these investments have not yielded proportional improvements in population-level outcomes, highlighting that financial input alone does not ensure greater system performance. Persistent challenges in workforce capacity, infrastructure, and care delivery efficiency continue to constrain the NHS’s ability to convert funding into measurable health gains, an issue also underscored in the 2024 Lord Darzi Report, which highlights the urgent need for system renewal to restore timely access, care coordination, and innovation [67]. 

#### Summary

The UK’s NHS is a centralized system designed to provide universal access to care. In practice, however, it is undergoing systemic deterioration. Long waiting times at all system levels, outdated infrastructure, and shortages of essential drugs and technologies have increasingly become sources of patient harm and public mistrust. As public provision falters, private-sector access has expanded, deepening inequalities in timeliness and quality of care, particularly along socioeconomic lines. While the NHS remains symbolically important, it can no longer be regarded as a model of effective and equitable healthcare delivery.

## Discussion

**Fig. 1:**
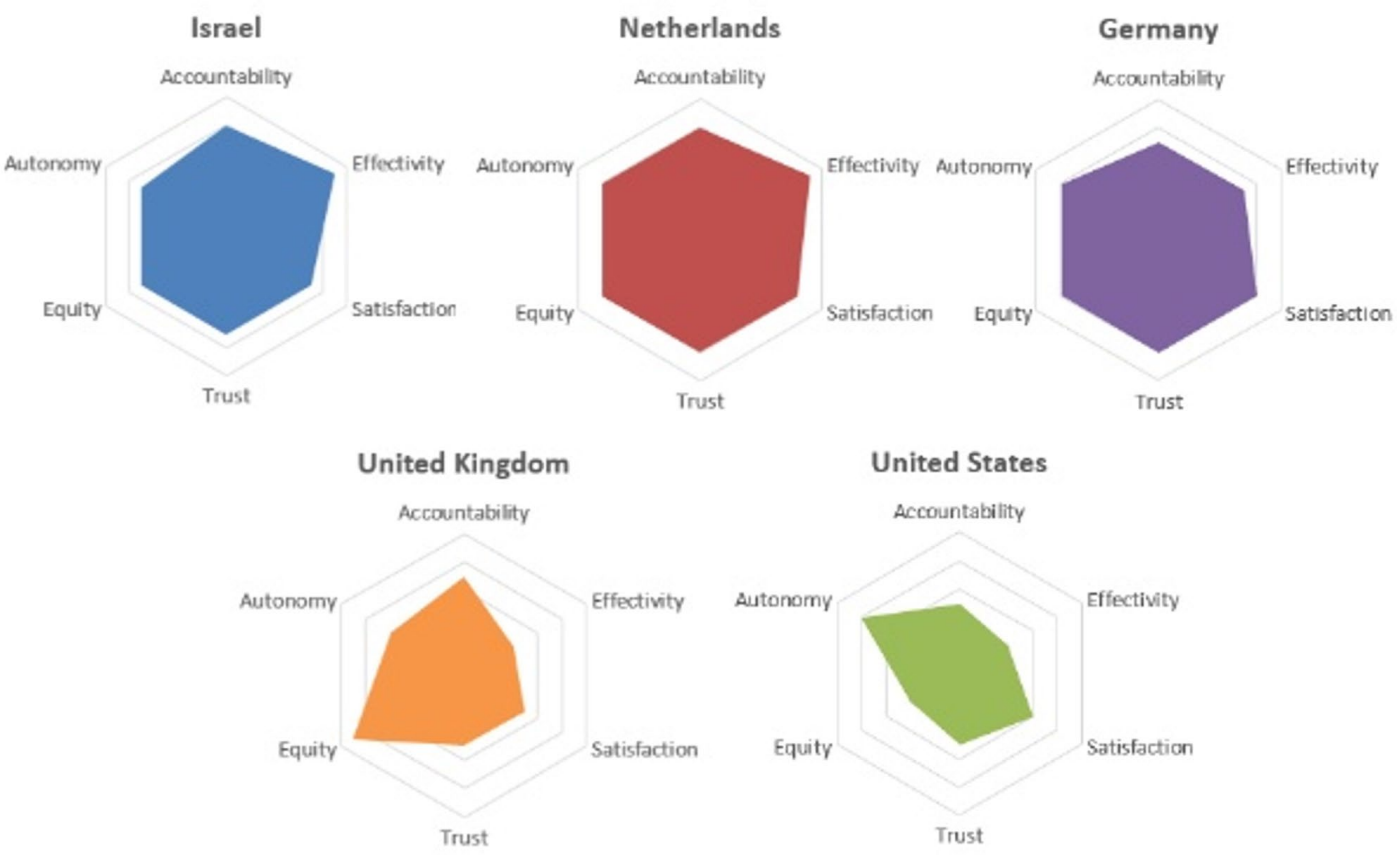
Radar chart comparing key dimensions of selected healthcare systems. This chart provides an abstract representation of how each country balances key factors in healthcare policy, offering a visual overview rather than precise rankings, highlighting each system’s relative strengths and weaknesses

This comparative analysis explored five high-income healthcare systems: Israel, the Netherlands, Germany, the US, and the UK, using a structured framework of six parameters: accountability, autonomy, equity, trust, satisfaction, and system effectiveness. While each health system demonstrates unique features shaped by its governance and cultural norms, cross-country comparison reveals recurrent tensions that inform how healthcare systems function and evolve. A summary of system performance across parameters is presented in Table 1; Fig. 1.

**Table 1:** A comparison of healthcare systems in Israel, the Netherlands, Germany, the United Kingdom, and the United States

Parameter	Indicator	Israel	Netherlands	Germany	United Kingdom	United States	OECD average
System overview/effectiveness	System type	Public–private (HMO)	Regulated competition	Statutory/private mix	Public (NHS)	Private with public programs	
	Health expenditure (% GDP)	7.4	10.2	12.7	11.3	16.6	9.2
	Average life expectancy at birth (years)	82.6	81.4	80.8	80.4	76.4	80.3
	Avoidable mortality rate (per 100,000)	141	161	195	222	336	237
	Physicians (per 1,000)	3.4	3.9	4.5	3.2	2.7	3.7
	Avoidable admissions (per 100,000)	440	318	728	403	725	463
Accountability	Model of oversight	Centralized (MoH, HMOs)	National framework agreements	Decentralized (federal-municipal)	Centralized (NHS)	Decentralized, mixed oversight	
	Population coverage (%)	100	99.9	99.9	100	91.3	97.9
Autonomy	Qualitative summary	Structured, gatekeeping, implemented reforms	High with selective contracting	High with free provider choice	Limited by gatekeeping and geography	High, limited by affordability	
Equity	Out-of-pocket expenditure (% of health expenditure)^a^	32.1	9.3	11	Minimal	11.1	
	Qualitative summary	Universal, but socio-geographic gaps	Universal, some cost barriers	Universal, dual system inequity	Universal, capacity-based access failures	No universal coverage, socioeconomic disparities	
Satisfaction	Satisfaction (%)	69	83	85	67	75	66.8
Trust	Qualitative summary	High trust in HMOs, low in financing	Strong institutional trust	High provider trust	Symbolic legitimacy, eroding under strain	Low institutional trust	
	Annual insurer switching (%)^a^	2–3%	6–7%	N/A	N/A	N/A	

Numerical data were retrieved from the 2023 OECD report [27] unless otherwise specified. GDP: gross domestic product; HMO: health maintenance organization; MoH: Ministry of Health; NHS: National Health Service; N/A: not applicable; OECD: The Organization for Economic Co-operation and Development. ^a^Data sources are mentioned in the manuscript

We emphasize several underlying tensions that challenge the stability of modern healthcare systems: the balance between accountability and autonomy; the accountability challenge in privately involved and decentralized systems; the coexistence of universal coverage with uneven access; and the complex relationship between system effectiveness and financial investment.

### Accountability and autonomy balance

One of the key insights from this analysis is that effective healthcare systems must carefully balance accountability and autonomy. Countries such as Israel and the Netherlands demonstrate that it is possible to maintain strong governmental supervision while enabling meaningful patient choice. In both systems, patients retain the ability to select providers, either directly (as in the Netherlands) or within structured frameworks (as in Israel’s HMOs), while regulatory mechanisms ensure transparency, equity, and efficiency. This balance supports high levels of satisfaction and system performance. In contrast, the UK illustrates the risks of prioritizing centralized accountability without sufficient patient flexibility. While the NHS is financially protective, limited autonomy, among other factors, may contribute to declining satisfaction and trust.

The gatekeeping model further exemplifies the tension between devotion to the patient and loyalty to the system. Designed to safeguard system resources and ensure care coordination, gatekeeping assigns the GP a dual role: patient advocate and steward of system efficiency. In systems like the UK and Israel, where GP referrals are typically required for specialist access, this structure enhances accountability but may limit perceived autonomy. In contrast, systems with fewer gatekeeping constraints, such as Germany or the US, allow more direct access but face challenges of care overuse, higher costs, and coordination gaps. These differences highlight that careful institutional design is needed to balance and reinforce both autonomy and accountability without undermining equity or sustainability.

### Accountability in privately involved and decentralized health systems

While the public-private distinction is often framed as a binary, the comparison between the Netherlands, Germany, and the US reveals that private-sector involvement can either support or undermine public goals, depending on how accountability is structured.

In the Netherlands, private for-profit insurers and providers operate within a tightly regulated statutory framework that includes mandatory coverage, benefit standardization, risk equalization, and outcome monitoring. These mechanisms ensure that private delivery remains aligned with public health objectives, demonstrating how central regulation can coexist with decentralized financing and delivery to support consistent performance.

Germany offers an example of a mixed and decentralized model with functional accountability. Publicly owned hospitals are jointly governed by state (Länder) governments, which fund infrastructure, and municipal governments, which oversee day-to-day clinical management. This dual structure reflects the geographic and demographic realities of a large federal country, enabling regionally responsive care while maintaining national standards.

In the US, dominant private ownership operates within a decentralized governance structure shaped by geographical, constitutional, historical, and cultural factors. While decentralization may be inevitable and beneficial in large, heterogeneous societies, weak national coordination results in inefficiencies and disparities.

This contrast highlights that accountability is not determined by ownership alone, but by design and enforcement of regulatory standards. Importantly, Centralized government control is not inherently superior. In countries with diverse and constitutionally decentralized structures, such as the US and Germany, governance through multiple levels and actors may be more aligned with institutional history and operational feasibility. In such settings, effective performance depends not on centralization, but on clear accountability, policy coherence, and coordination mechanisms tailored to national complexity.

As Israel’s healthcare system evolves, its hybrid model, characterized by strong statutory entitlements alongside growing private expenditure, will require more robust accountability mechanisms. The Dutch and German systems offer potential paths, demonstrating how private-sector delivery can coexist with equitable access, provided that public oversight remains comprehensive, transparent, and enforceable.

### Autonomy and system sustainability

While patient autonomy is a core value in many health systems, unstructured choice can undermine sustainability when not supported by coordination and resource planning. The Netherlands, Germany, and the US offer high formal autonomy, allowing individuals to choose among insurers, plans, and providers, but this is often constrained in practice by financial barriers, narrow networks, or workforce shortages. In contrast, Israel and the UK employ more structured autonomy: referrals are typically required for specialist care, and provider options are shaped by geography or organizational contracts. However, this does not mean a lack of choice. In Israel, reforms such as the Health Information Mobility Law and improved HMO switching mechanisms have expanded meaningful autonomy within a coordinated framework. Ultimately, healthcare systems must balance respect for the patient’s choice with mechanisms that promote equity, continuity, and efficient resource use.

### Universal coverage and uneven access

Most health systems in this study formally guarantee universal access to care. In practice, however, equitable and timely service delivery varies widely across and within countries. Universal coverage translates into real-world experience through differences in financing, infrastructure, workforce capacity, and regulatory effectiveness. The gap between entitlement and delivery remains a core challenge, even in well-resourced settings.

In the UK, care is provided free at the point of use through the NHS, but prolonged system strain has led to widespread delays, service failures, and growing public dissatisfaction. Access breakdowns, including long ambulance response times, overcrowded emergency departments, and staff shortages, suggest that the NHS can no longer reliably meet care obligations. These are not isolated problems, but structural consequences of a centrally managed, politically budgeted system that struggles to align entitlement with capacity. The NHS illustrates that narrowly defining equity as uniform access to publicly funded services may obscure deeper disparities in actual care. In resource-constrained settings, equal allocation can mean insufficient or delayed treatment for all, rather than prioritizing clinical need, undermining both effectiveness and ethical fairness.

The Netherlands and Germany provide near-universal coverage, but emerging challenges such as regional workforce gaps and dual-tier insurance models introduce inequities in access and timeliness. Israel’s national basket guarantees broad coverage, yet growing reliance on supplementary insurance and out-of-pocket spending has raised concerns about socioeconomic equity. In the US, coverage remains incomplete, and access varies widely by income, employment, and geography, reflecting both structural diversity and the absence of universal entitlement.

Even among geographically compact, high-income countries, regional disparities in access persist. While both Israel and the Netherlands are small in size, such disparities appear more pronounced in Israel. These reflect differences in primary care availability, infrastructure planning relative to population distribution, and uneven policy implementation across districts.

These findings underscore that universal coverage alone is insufficient. Equity and effectiveness depend not only on entitlement, but also on delivery capacity, infrastructure distribution, and policy mechanisms that translate coverage into timely, appropriate care.

### System effectiveness and financial investment

Another important lesson is that higher spending does not necessarily guarantee healthcare efficiency or quality of care. The case of the US underscores this point, as it spends a disproportionately high percentage of its GDP on healthcare without yielding the expected improvements in clinical outcomes. Despite substantial investment, the US continues to experience lower life expectancy and higher avoidable mortality rates compared to other countries like Israel and the Netherlands, which spend significantly less. Similarly, in the UK, recent increases in healthcare expenditure have not been translated into proportional improvements in system performance, in part due to persistent capability constraints and workforce shortage. These cases highlight that financial input alone is insufficient. The experiences of Israel and the Netherlands suggest that high performance can be achieved with more modest spending, provided that systems are well-organized and responsive to population needs.

### Lessons learned

Overall, the comparative findings suggest that well-regulated social health insurance models, such as those in the Netherlands, Germany, and Israel, may offer a more balanced approach to achieving equity, autonomy, and system performance than either loosely regulated private-market systems or highly centralized public models. These systems demonstrate how structured supervision and regulated competition can coexist with universal coverage and sustained public trust.

While structural and contextual differences between systems are substantial, several observations bear relevance for the Israeli health system. First, Israel’s core strengths – universal coverage, a comprehensive benefit basket, and relatively low out-of-pocket spending for essential services reflect its statutory foundations and the integration of health funds with service delivery. However, growing dependence on supplementary insurance and private spending for non-core services threatens to erode equity and system coherence. Second, the Netherlands illustrates how regulated private markets can promote both efficiency and equity, provided they are supported by robust oversight, transparent benefit design, and risk equalization. This model is particularly relevant as Israel’s supplementary insurance market expands, highlighting the need for clear accountability mechanisms and regulatory boundaries to prevent inequities and preserve public trust. Third, Israel should draw lessons from the Netherlands’ success in minimizing regional disparities, compared to its own more uneven geographic access. Finally, the UK experience serves as a caution against equating formal entitlement with real-world equity. Persistent access delays and strained service delivery underscore the importance of capacity planning, workforce investment, and timely access, all critical to maintaining both high public confidence and sufficient system performance.

### Limitations

This analysis is subject to several limitations. First, international comparisons are constrained by differences in data sources and measurement standards. While the parameter framework allows structured comparison, it remains interpretive and may oversimplify complex institutional realities. Second, as a cross-sectional comparison, this analysis cannot establish causality between system characteristics and health outcomes or overall performance. Additionally, contextual factors, such as sociopolitical, cultural, demographic, or economic dynamics, shape system performance in ways that are not fully captured by this framework. Comparative interpretations must also account for population size, geographic scale, and social heterogeneity, which may influence outcomes independently of system structure. Finally, the scope of the study focused on high-income countries, limiting generalizability to other settings. These limitations highlight the need for caution in generalizing conclusions and underscore the importance of contextualizing cross-country findings.

## Conclusions

This analysis highlights enduring structural tensions in health system design: autonomy versus accountability, public versus private provision, decentralization versus fragmentation, and universal coverage versus real-world access. While no system resolves these trade-offs entirely, well-regulated social health insurance models, such as those in the Netherlands and Germany, appear to manage them more consistently, balancing patient choice with equity, oversight, and sustainability.

For Israel, the findings carry both caution and guidance. Its statutory foundations remain strong, but growing reliance on supplementary insurance and regional disparities risk eroding core equity principles. Experience from the Netherlands and Germany suggests that effective oversight, clear regulatory boundaries, and attention to regional equity can help Israel preserve public trust while navigating a hybrid public-private model.

Universal coverage is a necessary foundation, but not a guarantee of effective or fair care. Health system performance ultimately depends on how competing priorities are coordinated, regulated, and financed in practice. A structured comparative framework, though interpretive, can help reveal critical tensions and support more resilient and equitable reform across diverse national contexts.

## Data Availability

No datasets were generated or analysed during the current study.

## Abbreviations

ACA: Affordable Care Act
COVID: Coronavirus disease
EU: European Union
GDP: Gross domestic product
GP: General practitioner
HMO: Health maintenance organization
NHS: National Health Service
OECD: The Organization for Economic Co-operation and Development
SHI: Statutory health insurance
UK: United Kingdom
US: United States
VA: Veteran Affairs
